# Off-target effects of plasmid-transcribed shRNAs on NFκB signaling pathway and cell survival of human melanoma cells

**DOI:** 10.1007/s11033-013-2817-7

**Published:** 2013-10-30

**Authors:** Kavita Ramji, Dorota Weronika Kulesza, Salem Chouaib, Bozena Kaminska

**Affiliations:** 1 Laboratory of Transcription Regulation, Department of Cell Biology, The Nencki Institute of Experimental Biology, 3 Pasteur Str., 02-093 Warsaw, Poland; 2 Postgraduate School of Molecular Medicine, Villejuif, France; 3 U753 INSERM, Institute Gustave Roussy, Villejuif, France

**Keywords:** Transcription factor STAT3, STAT3-NFκB crosstalk, Cancer cell survival, RNAi

## Abstract

Signal transducer and activator of transcription 3 (STAT3) and nuclear factor kappa-light-chain-enhancer of activated B cells (NFκB) are transcription factors involved in cell survival, inflammation and metastasis. Constitutively activated STAT3 is found in many cancers, including melanoma. To study the crosstalk between STAT3 and NFκB signaling and its role in regulation of cancer cell survival, we used RNA interference (RNAi) to down-regulate STAT3 expression in human melanoma cells. RNAi strategies including double-stranded RNA, small interfering RNA (siRNA), short hairpin RNA (shRNA) and microRNA are widely used to knock down disease-causing genes in a targeted fashion. We found that shRNAs up-regulate non-specific NFκB activity, while siRNA directed against STAT3 specifically increase NFκB activity. The basal survival of melanoma cells is unaffected by STAT3 knockdown—likely due to activation of pro-survival NFκB signaling. Whereas, owing to off-target effects, plasmid-transcribed shRNA affects melanoma survival. Our data show that shRNA-mediated gene silencing induces non-specific or off-target effects that may influence cell functions.

**Electronic supplementary material:**

The online version of this article (doi:10.1007/s11033-013-2817-7) contains supplementary material, which is available to authorized users.

## Introduction

Clinical and experimental studies in the past provide compelling evidence for the important role of aberrant STAT3 signaling in cancer [1, 2]. Phosphorylation of STAT3 at tyrosine 705 leads to its dimerization, nuclear translocation, DNA binding and gene transcription. STAT3 is implicated in the regulation of many genes that mediate survival, cell-cycle progression, invasion, immune responses and angiogenesis [3–6]. Persistent activation of STAT3 occurs with high frequency in human cancers [7]. Blocking STAT3 activity using techniques such as: antisense, RNA interference, dominant negative STAT3 and small molecular inhibitors reduced cancer cell growth, invasion, and tumor size in vivo and increased apoptosis in vitro [8–13].

Nuclear factor-kappa B (NFκB) is another transcription factor that regulates many genes involved in immune and inflammatory responses. Studies exposed NFκB as a survival factor and blocking it’s activity promoted apoptosis in cancer cells and reduced resistance to chemotherapeutic drugs [14]. Cross-talk between STAT3 and NFκB signaling pathways is unclear and maybe cell type/genetic background specific. NFκB activity in the cells can be gauged by measuring the level of phosphorylated IκBα (P-IκBα). A study showed increased P-IκBα in lipopolysaccharide-induced *Stat3* ^−/−^ dendritic cells, suggesting that Stat3 may inhibits NFκB activity under normal conditions [15]. On the other hand, maintaining NFκB activity in both cancer cells and tumor-associated hematopoietic cells requires Stat3, as it prolongs NFκB nuclear retention [16]. Interactions between NFκB and phosphorylated STAT3 also contribute to cell survival in B cell lymphomas [17] and head and neck squamous cell carcinomas [18]. STAT3 and NFκB cross-talk includes: cooperation of these factors at gene promoters/enhancers; NFκB dependent expression of STAT3 inhibitors; and participation of STAT3 in the negative regulation of NFκB in inflammatory cells [19, 20]. Despite the versatile and occasionally antagonistic interactions, NFκB and STAT3 combine forces in development and progression of colon, gastric and liver cancers [21].

We examined the effects of silencing STAT3 expression on the NFκB activity in melanoma cells. We discovered non-specific or off-target effects in two commonly used control short hairpin RNA (shRNAs) that increased NFκB activity while silencing STAT3 expression with small interfering RNA (siRNA) resulted in the specific increase of the NFκB activity. STAT3 depletion did not affect basal survival of melanoma cells but STAT3 silencing using specific shRNA affected the survival of melanoma cells. This suggests that shRNA-mediated gene silencing may induce non-specific or off-target effects such as NFκB activation, which in turn may affect cell survival.

## Materials and methods

### Cell cultures and treatment

Human metastatic melanoma WM239, WM902 and non-metastatic T1 cells were cultured in RPMI media supplemented with 10 % fetal bovine serum (Gibco, MD, USA) and antibiotics (50 U/ml penicillin, 50 μg/ml streptomycin and 1 % sodium pyruvate) in a humidified atmosphere of CO_2_/air (5/95 %) at 37 °C (Heraeus, Hanau, Germany). Cells were transfected with a plasmid DNA or siRNA 18 h after plating.

### Western blot analysis

Whole-cell lysates were prepared as previously described [22]. Cells were collected in lysis buffer with protease inhibitors (20 mM Tris–HCl, 137 mM NaCl, 25 mM β-glicerophosphate, 2 mM NaPPi, 2 mM EDTA, 1 mM Na_3_VO_4_, 1 % Triton-X, 10 % glycerol, 0.5 mM DTT, 1 mM PMSF, 15 μg/ml aprotinin and leupeptin, 2 mM benzamidine). Protein samples were resolved by SDS-PAGE and transferred to nitrocellulose membranes (Amersham). Antibodies recognizing phospho and total forms of STAT3, IκB and cleaved PARP were obtained from Cell Signaling, USA. The membranes were stripped and re-probed with horseradish peroxidase-conjugated anti-β-Actin antibody (Sigma-Aldrich, Saint Louis, MO, USA).

Membranes were incubated with primary antibodies diluted in TBS-T (10 mM Tris/HCl, pH 8.0, 0.12 M NaCl, 0.1 % Tween-20 and 0.05 % sodium azide) containing 5 % skimmed milk. Antibody recognition was detected with the respective secondary antibody either anti-mouse IgG or anti-rabbit IgG linked to horseradish peroxidase (Cell Signaling, USA). Immunocomplexes were visualized using enhanced chemiluminescence detection system (ECL, Amersham).

### STAT3 silencing shRNA expressing vectors and siRNAs

Four complementary oligonucleotides encoding STAT3 shRNA with *Bam*H1 and *Hin*dIII overhangs were designed to interfere with the expression of human STAT3 mRNA. The single-stranded oligonucleotides were purchased from Sigma Aldrich (Sigma Life Science). The sequences of shSTAT3 were: shStat3.1 [23] 5′-GATCCGCAGCAGCTGAACAACATG TTCAAGAGACATGTTGTTCAGCTGCTGCTTTTTGGAAA-3′ and 5′-AGCTTTTCCAAAAAAGCAGCAGCTGAACAACATGTCTCTTGAA CATGTTGTTCAGCTGCTG CG -3′ shStat3.2 [24] 5′-GATCCGCTTCAGACCCGTCAACAAA TTCAAGAGATTTGTTGACGGGTCTGAAGTTTTTTGGAAA-3′ and 5′-AGCTTTTCCAAAAAACTTCAGACCCGTCAACAAATCTCTTGAA TTTGTTGACGGGTCTGAAGCG-3′

Forward and reverse oligonucleotides double-stranded oligomers were prepared by incubation in 0.1 M NaCl for 3 min at 94 °C, followed by slow cooling to 37 °C. The annealed DNA was ligated with linearized pSilencer 2.0-U6 (Ambion) at *Bam*HI and *Hin*dIII sites. The sequence of resulting plasmids: pSilencer-STAT3.1_hs and STAT3.2_hs was further verified by direct sequencing. The plasmid DNA was isolated with Qiagen Plasmid Maxi Kit.

STAT3 siRNA which is a pool of three target-specific 20–25 nt siRNA designed to knock down gene expression was obtained from Santa Cruz Biotechnology (sc-29493). Control siRNA-A was obtained from Santa Cruz Biotechnology (sc-37007), is a non-targeting 20–25 nt siRNA designed as a negative control.

sc-29493: Stat3 siRNA (h) is a pool of 3 different siRNA duplexes:

sc-29493A:

- Sense: GAGACAUGCAAGAUCUGAATT
- Antisense: UUCAGAUCUUGCAUGUCUCTT

sc-29493B:

- Sense: GGAUCCCGGAAAUUUAACATT
- Antisense: UGUUAAAUUUCCGGGAUCCTT

sc-29493C:

- Sense: CCUCUCUGCAGAAUUCAAATT
- Antisense: UUUGAAUUCUGCAGAGAGGTT

### Transfection and gene reporter assay

WM239, WM902 and T1 melanoma cells were plated in 12-well plates in a complete medium (DMEM, 10 % FBS, sodium pyruvate and antibiotics) at a density of 8 × 10^4^ cells/well. Twenty four hours after seeding melanoma cells were transfected by electroporation with 2.5 μg/well of the indicated plasmid or the indicated siRNA at the final concentration of 150 nM using the Amaxa and Cell Line Nucleofector Kit V (Lonza), according to the manufacturer’s protocol. In the preliminary experiment, the cells were co-transfected with the reporter plasmid carrying a firefly luciferase gene under a promoter consisting of multiple binding sites for NFκB (NF-κB p65 cis-Reporting System, Stratagene-Agilent Technologies Company, CA, USA) and EGFP vector (pEGFP-N1 Vector Information PT3027-5, GenBank Accession #U55762 Catalog #6085-1) coding for green fluorescence protein and analyzed after 48 h with fluorescent microscopy to determine efficacy of transfection. siRNA transfection efficiency was assessed by co-transfecting the cells with siGlo Red siRNA (557–570 nm) from Thermo Scientific Dharmacon and control and STAT siRNA and analysed after 48 h using flow cytometry analysis (FACSCalibur using CellQuest software). After transfection cells were plated into 24-wells plate at a density of 2 × 10^5^/well and incubated for 48 h.

To study NFκB activation, the cells were cotransfected with 2.5 μg of the reporter plasmid carrying a firefly luciferase gene under promoter consisting multiple of binding sites for NFκB (NF-κB p65 cis-Reporting System, Stratagene-Agilent Technologies Company, CA, USA) and p-Super vector (VEC-PBS-0002 OligoEgine) and PCMV6-XL5(Origene) using Lipofectamine™ 2000 Reagent (Invitrogen, Carlsbad, CA). Twenty four hours after transfection the cells were lysed in a passive lysis buffer and luciferase activity was measured using a Promega luciferase kit.

### NFκB DNA binding activity ELISA

NFκB DNA binding activity was determined using TransAM^®^ NFκB p65 Kit from Active Motif (Carlsbad, CA, USA). Each kit includes a 96-stripwell plate in which multiple copies of a specific double-stranded oligonucleotide have been immobilized. Nuclear protein extract were isolated and incubated according to the manufacturer’s protocol to allow the activated transcription factor to bind the oligonucleotide at its consensus binding site. Two μg of nuclear extract was incubated for 1 h with the primary antibody recognizing an active form of the transcription factor followed by 1 h incubation with the secondary antibody allowed quantification of the amount of NFκB.

### Analysis of cell viability by MTT assay

The number of living cells was evaluated with an MTT metabolism test. Melanoma cells were cultured on 24-wells plates as described. After specific treatments, MTT (Thiazolyl Blue Tetrazolium Bromide, Sigma-Aldrich, Munich, Germany) stock solution was added to a final concentration of 0.5 mg/ml. After 4 h of incubation at 37 °C a lysis buffer containing 20 % SDS and 50 % DMF was added and optical densities in extracts were determined at 570 nm using a scanning multiwell spectrophotometer (Thermo labsystem Multiscan EX).

### TUNEL staining for DNA fragmentation in situ

Cells were transfected with control and targeted siRNA or shRNA, and plated on glass cover slips in 24 well plates. After transfection (48 h) the cells were fixed using 4 % paraformaldehyde and permeabilised using 0.1 % Triton X-100 in 0.1 % sodium citrate. The cells were then stained with the TUNEL mixture using the manufacturer’s guide. The cover slips were then mounted onto glass plates using Dako Flourescent medium (Dako North America Inc). Samples were then analysed under a fluorescence microscope using a detection wavelength of 515–565 nm. TdT-mediated dUTP-biotin nick end labeling (TUNEL) (Roche, USA) was conducted to identify apoptotic cells. The percentage of TUNEL-positive cells was defined as the number of TUNEL-stained cells divided by the total number of cells seen within bright field. At least 600 cells in 3 randomly selected fields of view from each well were counted for 3 independent experiments.

### Quantitative evaluation of gene expression

RNA was extracted using the RNeasy Mini Kit from Qiagen. The quality and concentration of RNA was checked using Nanodrop 2000 spectrophotometer from Thermo Scientific. Total RNA from each sample was converted into cDNA using the SuperScript First-Strand Synthesis System (Invitrogen) for PCR according to manufacturer’s instructions. TaqMan/Syber green real time quantitative reverse transcription-PCR (qRT-PCR) was used to measure the mRNA expression levels for *STAT3* and *IRF7.* The primers sequences were: *IRF7*: GCGGTGCAAGAGCCCAGCCC - CGTGCAGCTCGGGTGTCCCA; *STAT3*: ATTGCCCGGATTGTGGCCCG - CTCCGTCACCACGGCTGCTG; and *18S* AACGAACGAGACTCTGGCATG - CGGACATCTAAGGGCATCACA; The amount of target mRNA was normalized to the expression level of the 18S rRNA amplified from the same sample. The relative quantification of gene expression was determined with ABI PRISM 7700 using the comparative CT method.

### Statistical analysis

To assess the differences between specifically manipulated cells and the respective controls, data were analyzed by Student’s *t*-test using Microsoft Excel software and are presented as mean ± S.D. *P < 0.05, **P < 0.01, ***P < 0.001 were considered statistically significant.

## Results

### Plasmid transcribed shRNAs nonspecifically induce NFκB activity in melanoma cells

**Fig. 1:**
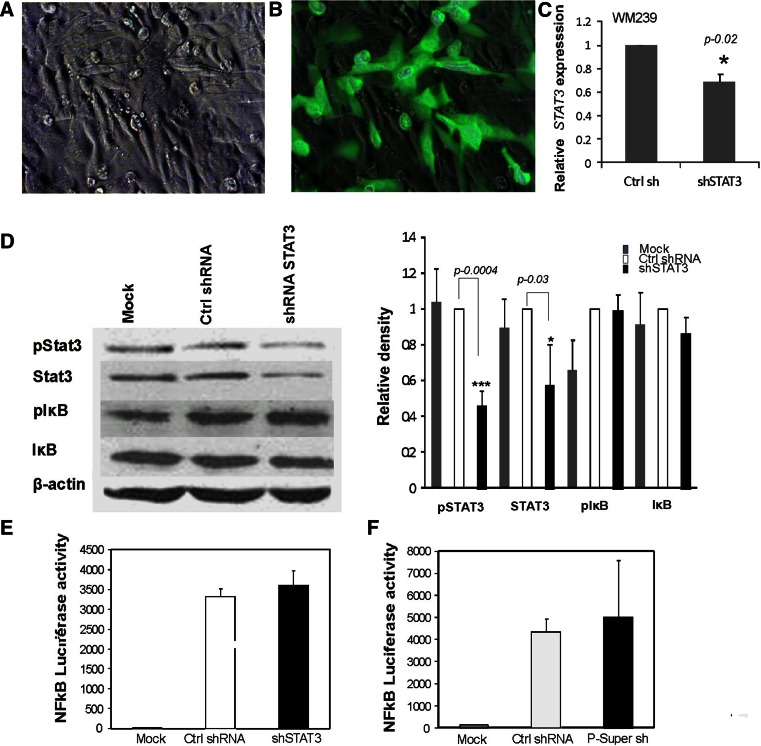
Plasmid transcribed shRNAs nonspecifically induce NFκB activity in melanoma cells. **a**–**b** Representative pictures of WM239 melanoma cells 48 h after co-transfection with p-Silencer encoding STAT3 shRNA and EGFP plasmids. Using phase contrast (**a**) and fluorescence microscopy (**b**), transfection efficiency was evaluated as ~65 %. **c** The level of STAT3 silencing at the RNA expression level was evaluated with qPCR and related to STAT3 expression in the control shRNA transfected cells. **d** The levels of phosphorylated and total STAT3 and IκB proteins in cells expressing control shRNA or STAT3 specific shRNAs were examined by Western blotting. Immunoblots were re-probed with an antibody recognizing β-actin to ensure equal loading. Similar results were obtained in four independent experiments. Densitometry of immunoblots using Image J was performed. The analysis of the Western blots was done using β-actin as the loading control. **e**–**f** Increase of the NFκB transcriptional activity in WM239 melanoma cells transfected with plasmids encoding shRNA against STAT3 or control shRNAs. The cells were co-transfected with plasmids encoding STAT3 shRNA and NFκB reporter plasmids using Lipofectamine 2000. Luciferase activity was determined after 48 h using a dual light luciferase assay and normalized to the protein content determined in the sample. The results indicated that the activation of NFκB transcriptional activity is not specific for shRNA against STAT3. Two commercial control shRNAs cause similar up-regulation of NFκB-driven transcription (**f**). The results of three separate experiments are presented as mean ± S.D.

To study the interactions between STAT3 and NFκB in melanoma cells, STAT3 expression was silenced with the plasmid transcribed, specific shRNAs. In preliminary experiments, we used two previously described shRNAs [23, 24]. The most effective shRNA was used for further experiments. First, WM239 melanoma cells were co-transfected with the EGFP plasmid and plasmids coding for control shRNA (shNeg) and were analyzed after 48 h with light and fluorescence microscopy. Transfection efficiency was evaluated as ~65 % (Fig. 1a, b). The level of silencing at the RNA level is shown (Fig. 1c). Immunoblots show effective silencing of STAT3 expression at the protein level 48 h after transfection with a densitometric analysis (Fig. 1c, d). The results of three separate experiments showed the increase of NFκB transcriptional activity in melanoma cells transfected with plasmids coding for STAT3 specific and control shRNA (Fig. 1f). Activation of the transcription factor NFκB is controlled at multiple levels and requires phosphorylation, ubiquitination and degradation of its inhibitor IκB that allows NFκB to translocate to the nucleus and to induce specific gene expression [25]. The level of phosphorylated (p)IκBα was increased in melanoma cells transfected with plasmids coding for shSTAT3 and control shRNA suggesting off target activation by shRNAs (Fig. 1d). These results indicate that the activation of NFκB transcription is not specific for shRNA against STAT3.

To assess if the off target effect was specific for the p-Silencer based plasmid, melanoma cells were co-transfected with the NFκB-Luc plasmid and two widely used plasmids coding for control shRNAs (p-Silencer Neg and p-Super Ctr). The increase in the NFκB activity was also found when the commonly used p-Super based control shRNA was used (Fig. 1g).

### STAT3 knockdown with siRNA leads to NFκB activation in human melanoma cells

**Fig. 2:**
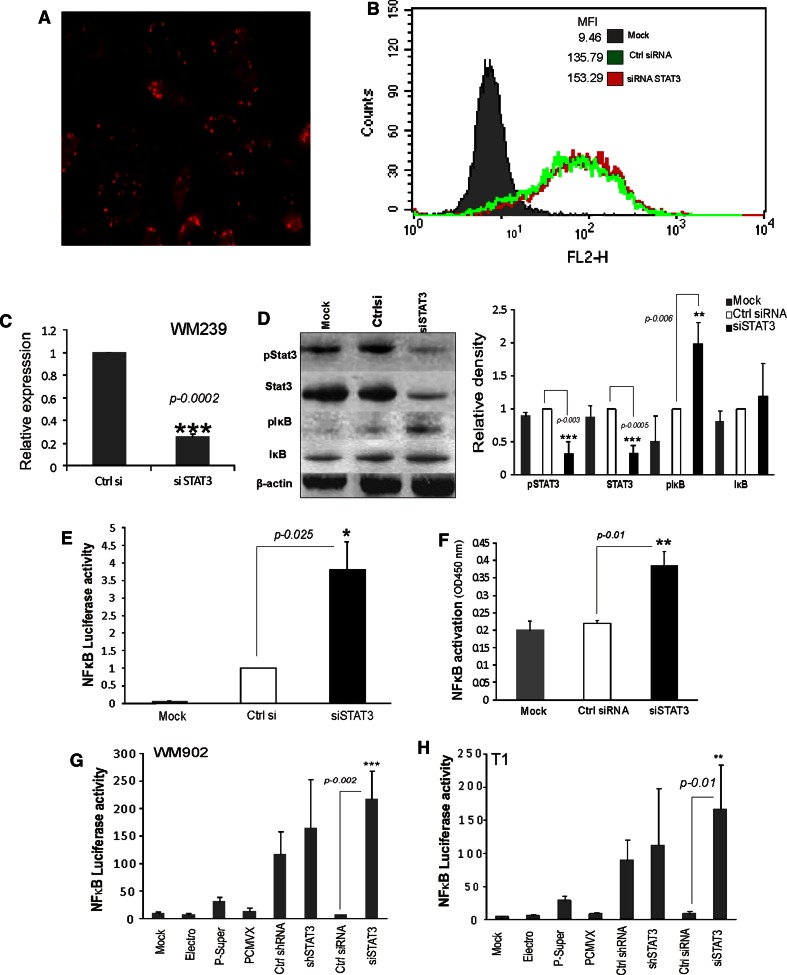
STAT3 knockdown with siRNA leads to NFκB activation in human melanoma cells. **a**–**b** Representative pictures of WM239 melanoma cells 48 h after transfection with siRNA labeled with Rhodamine (siGlo) using AMAXA electroporation. Using fluorescence microscopy (**a**) and flow cytometry analysis (**b**) the efficacy of siRNA transfection was determined as 84 %. **c** Effective STAT3 knockdown in WM239 cells. The *STAT3* mRNA level was determined using qPCR and was related to its level in control siRNA transfected cells. **d** The levels of phosphorylated and total STAT3 and IκB proteins in cells transfected with control or STAT3 specific siRNA were examined by Western blotting. Immunoblots were re-probed with an antibody recognizing β-actin to ensure equal loading. Similar results were obtained in three independent experiments. The *right panel* shows quantification of the Western blots from three experiments using Image J with β-actin as the loading control. **e** The increase of the NFκB transcriptional activity in melanoma cells transfected with STAT3 specific siRNA. Cells growing onto 24-wells plates were co-transfected with the NFκB-luc plasmid and control or STAT3 siRNA using AMAXA electroporation. The luciferase activity was measured 48 h after transfection. The *bars* indicate mean values of luciferase activity in the mock transfected cells and cells transfected with the control or STAT3 siRNA. Data are presented as mean ± S.D. from three experiments, each in duplicate. **f** Evaluation of NFκB DNA binding by ELISA. Cells (1 × 10^7^ cells/per group) were mock transfected or were co-transfected with a control or STAT3 siRNA using AMAXA electroporation. Cell nuclei extracts were collected 48 h after transfection and 2 μg of nuclear extract was subjected to an NFκB DNA binding assay (Active Motif—TransAM^®^ NFκB Family Kit, Carlsbad, USA). The NFκB -ELISA assay results demonstrated an increase in the NFκB binding to DNA after silencing the expression of STAT3. Data are presented as mean ± S.D. from three experiments. **g**–**h**. STAT3 knockdown with siRNA induces specifically NFκB-Luciferase activity in WM209 and T1 melanoma cell lines. The NFκB-Luciferase activity measured in WM209 and T1 cells untreated (mock), treated with electroporation only or in cells transfected with two empty plasmids such as p-Super and PCMV6-XL5, or plasmids coding for shRNAs or siRNAs. An increase of the NFκB transcriptional activity was observed in melanoma cells transfected with plasmids encoding shRNA against STAT3 and control shRNAs and siRNA against STAT3 and control. Electroporation itself or transfection with empty plasmids do not induce NFκB activation. Data are presented as mean ± S.D. from three experiments

Consequently, we studied the effects of siRNA-mediated knockdown of STAT3 on NFκB activity. Figure 2a shows that human melanoma cells were effectively transfected with siRNA labeled with Rhodamine (siGlo) 48 h after transfection. The transfection efficacy was established as 84 % using fluorescence microscopy and flow cytometry (Fig. 2a, b). STAT3 siRNA was a pool of three target-specific siRNA designed to knock down gene expression. There was profound silencing of STAT3 expression at the mRNA and protein level 48 h after transfection in cells transfected with the siRNA against STAT3, compared with the control siRNA (Fig. 2c, d). Similarly there was an increase in the level of pIκBα in cells transfected with the siRNA against STAT3, but not with the control siRNA supporting the fact that there was increase in the NFκB-luciferase activity (Fig. 2e, f) only after STAT3 was knocked down. The results showed that the control siRNA did not significantly increase the NFκB-luciferase activity. The results were corroborated by a NFκB DNA binding ELISA assay that measures how activated NFκB in the cell extract binds to the consensus-binding site. The NFκB DNA binding ELISA results indicated that after silencing the expression of STAT3, there was an increase in the NFκB binding to DNA (Fig. 2f). These results confirmed our data collected using NFκB-luciferase activity and the pIκBα Western blotting.

In order to assess if STAT3 knockdown induces similar changes in the NFκB-luciferase activity in different cell lines, we measured NFκB-luciferase activity in WM902 and T1 cell lines (Fig. 2g, h). The results of three separate experiments showed the increase of NFκB transcriptional activity in melanoma cells transfected with plasmids coding for STAT3 specific control shRNA and siRNA against STAT3. The results showed that the control siRNA did not significantly increase NFκB-luciferase activity. There was no increase in the NFκB-luciferase activity observed in cells after electroporation and in cells transfected with empty plasmids including p-Super vector and PCMV6-XL5. The silencing effects in cell lines were determined with qPCR and considerable reduction in the level of STAT3 mRNA was observed (supplementary Fig. 1a).

The off-target effect from shRNA transfection could be due to activation of interferon-related responses when expressed in the cells. We determined the level of *IRF7* (interferon response factor 7) gene expression in shRNA or siRNA transfected cells (supplementary Fig. 1a). There was an increase in the expression of *IRF7* after transfecting the cells with control and STAT3 specific shRNA, when compared to cells transfected with siRNA. Altogether, the results indicate that siRNA would be a better tool for gene silencing to study the cross talk between STAT3 and NFκB in transient transfection experiments.

### STAT3 knockdown with specific shRNA, but not siRNA, reduces cell survival of melanoma cells

**Fig. 3:**
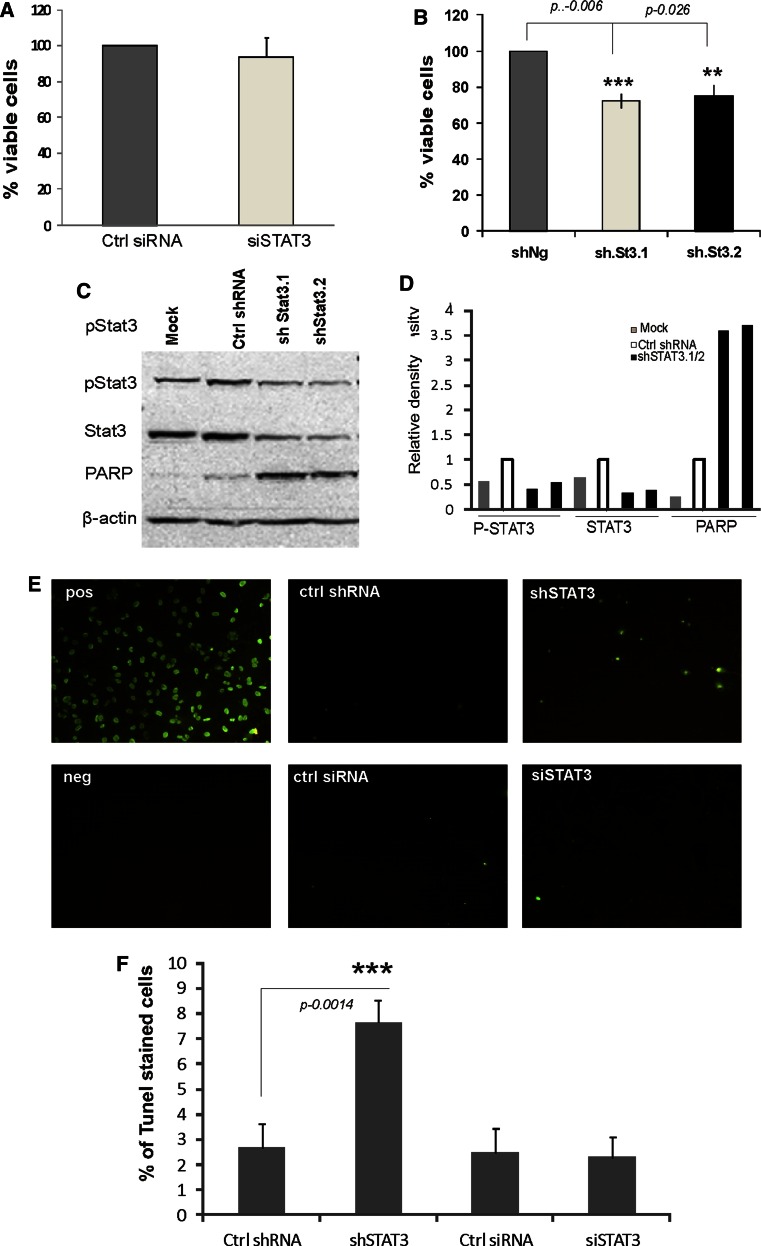
STAT3 knockdown with specific shRNA, but not siRNA, reduces cell survival and induces DNA fragmentation in melanoma cells. **a**–**b** Cells (1 × 10^7^ cells/per group) were mock transfected or were transfected with a control or STAT3 siRNA or plasmids coding for control or STAT3 specific shRNAs. Cell viability was determined using the MTT metabolism assay 48 h after the transfection of the indicated shRNA or siRNA. Measurements were related to respective controls (cells transfected with ctr siRNA or shRNA were taken as 100 %) and data represent mean ± S.D. from three experiments, each in triplicate. **c** STAT3 knockdown with two specific shRNAs results in induction of apoptotic cell death. The levels of phosphorylated and total STAT3 and cleaved PARP proteins in cells transfected with plasmids coding for control or STAT3 specific shRNA were examined by Western blotting. Immunoblots were re-probed with an antibody recognizing β-actin to ensure equal loading. Similar results were obtained in three independent experiments. **d** Quantification of the Western blots by using β-actin as the loading control using Image J. **e** DNA fragmentation occurs in cells transfected with STAT3 specific shRNA. TUNEL staining was performed on cells 48 h after transfection and then observed using a fluorescence microscope. TUNEL staining is shown in *green*. In the *top right* is the positive control with cells incubated with Dnase1 and the negative control below are cells labeled with Label solution (without terminal transferase). **f** The percentage of TUNEL-positive cells was defined as the number of TUNEL-stained cells divided by the total number of cells seen with bright field. At least 600 cells in three randomly selected fields of view from each well were counted for three independent experiments

In order to assess cell viability upon silencing the expression of STAT3 with various tools, an MTT metabolism assay was carried out 48 h after transfection. The MTT results demonstrated that siRNA mediated STAT3 knockdown did not affect cell survival (Fig. 3a). In the contrary we observed reduction of cell viability in cells transfected with the plasmids coding for two different STAT3 shRNAs (Fig. 3b). Strong accumulation of the cleaved PARP, a hallmark of apoptotic cell death, was observed in those cells (Fig. 3c, d).

Apoptotic cells can be detected by terminal deoxynucleotidyl transferase (TdT)-mediated dUTP nick end labeling (TUNEL). TUNEL staining was used to detect DNA fragmentation, which is one of the hallmarks of apoptosis. We performed recommended controls both positive (a DNAase treatment) and negative (an enzyme omitted). As expected, the numbers of TUNEL-positive cells (stained with green fluorescence) markedly increased 48 h after silencing with shSTAT3 compared to control and siSTAT3 (Fig. 3e, f). These results show that silencing of STAT3 expression with shRNA affects cell viability, while even greater knock down of STAT3 expression with siRNA, does not impair basal cell survival.

## Discussion

In the present study we report two main observations: First, we identified NFκB activation as a novel off target effect of control plasmid transcribed shRNAs; secondly, we demonstrate that effective STAT3 silencing induces NFκB activation that may compensate for the role of STAT3 in tumor cell survival. RNAi based technologies have become an increasingly popular approach, but their usefulness is limited by the occurrence of unintended off-target effects. The off-target effects implicate that in addition to targeting the intended gene product, artificial shRNA/siRNAs can produce unspecific outcomes [26–28]. Even the most widely used control siRNA directed against GFP has been reported to have off-target effects and deregulate a set of endogenous genes in addition to GFP. The off-target effects were dependent on the amount of GFP siRNA transfected and were detected in a variety of cell lines [28]. Off-target effects may compromise the specificity of RNAi by down-regulating the expression of multiple mRNAs through microRNA-like targeting of the 3′ untranslated region. siRNA therapeutics may trigger microRNA-like silencing of many unintended targets in vivo complicating the interpretation of phenotypic effects and may potentially lead to unwanted toxicities [29, 30]. Many studies demonstrated the utility and efficacy of shRNAs in vitro and in vivo [31]. The shRNAs were found more potent than the artificial microRNA (miRNAs) in gene silencing in vitro and in vivo. However, greater off-target effects and interferon responses were induced by shRNAs than by their corresponding siRNAs [32, 33]. We have identified NFκB activation as a new type of cellular stress response triggered by two commonly used shRNAs. NFκB activation may offer an explanation for the off-target effects of RNAi-based therapeutics and suggests the requirement for very careful interpretation of experiments using shRNA to study cell survival, especially after transient transfection.

We report that STAT3 knockdown with specific shRNA in melanoma cells resulted in impairment of cell survival and induction of apoptotic cell death, as evident by accumulation of the cleaved PARP and as detected by TUNEL staining, biochemical hallmarks of apoptosis. In contrast to reduced survival of melanoma cells expressing STAT3 shRNA, the cells depleted of STAT3 by siRNA did not show reduction of cell survival that we had expected. We demonstrate an induction of the NFκB activity (verified with three different assays: NFκB-driven luciferase assay, NFκB DNA binding ELISA and detection of phosphorylated IκB) in melanoma cells depleted of STAT3. The increased activation of NFκB and subsequent up-regulation of some pro-survival genes could compensate for STAT3 deficiency. A network involving co-activation of NFκB and STAT3, their compensation for each other, influence on BAX/BCL-X_L_ expression and cell survival has been described in head and neck squamous cell carcinomas [34]. Some studies demonstrated reduced survival of murine melanoma B16 cells depleted of Stat3 by siRNA in vitro and in vivo [35, 36]. However, our data show that silencing of STAT3 expression with siRNA does not affect basal proliferation of human melanoma cells. It corresponds to our recent studies that demonstrate unaffected survival of C6 glioma cells with siRNA mediated Stat3 knockdown [37]. The reduced survival of melanoma cells depleted of STAT3 with shRNAs could be a cumulative result of STAT3 silencing and off targets effects. Our results also show NFκB activation as a novel off target effect of control plasmid transcribed shRNAs (frequently used as negative controls) in human melanoma cells. Since off-target effects seemingly cannot be avoided, this calls for cautiousness in interpretation of shRNA effects on cell functions in transiently transfected cells.

## Electronic supplementary material

Below is the link to the electronic supplementary material.

Supplementary Fig. 1. **a** Level of STAT3 silencing at the level of RNA were determined using qPCR and related to its levels in cells transfected with the control shRNA or siRNA in case of T1 cells. Data are presented as mean ± S.D. from 3 experiments. **b** Relative expression of *IRF7* after transfection of melanoma cells with both siRNA and shRNA against STAT3. Data are presented as mean ± S.D. from 3 experiments. The significant increase of *IRF7* expression was observed in cells transfected with shRNAs. (JPEG 147 kb)

## References

[1] Yu H, Kortylewski M, Pardoll D (2007). Crosstalk between cancer and immune cells: role of STAT3 in the tumour microenvironment. Nat Rev Immunol.

[2] Kortylewski M, Yu H (2008). Role of Stat3 in suppressing anti-tumor immunity. Curr Opin Immunol.

[3] Catlett-Falcone R, Landowski TH, Oshiro MM, Turkson J, Levitzki A, Savino R, Ciliberto G, Moscinski L, Fernandez-Luna JL, Nunez G, Dalton WS, Jove R (1999). Constitutive activation of Stat3 signaling confers resistance to apoptosis in human U266 myeloma cells. Immunity.

[4] Masuda M, Suzui M, Yasumatu R, Nakashima T, Kuratomi Y, Azuma K, Tomita K, Komiyama S, Weinstein IB (2002). Constitutive activation of signal transducers and activators of transcription 3 correlates with cyclin D1 overexpression and may provide a novel prognostic marker in head and neck squamous cell carcinoma. Cancer Res.

[5] Puthier D, Bataille R, Amiot M (1999). IL-6 up-regulates mcl-1 in human myeloma cells through JAK/STAT rather than ras/MAP kinase pathway. Eur J Immunol.

[6] Fukada T, Hibi M, Yamanaka Y, Takahashi-Tezuka M, Fujitani Y, Yamaguchi T, Nakajima K, Hirano T (1996). Two signals are necessary for cell proliferation induced by a cytokine receptor gp130: involvement of STAT3 in anti-apoptosis. Immunity.

[7] Buettner R, Mora LB, Jove R (2002). Activated STAT signaling in human tumors provides novel molecular targets for therapeutic intervention. Clin Cancer Res.

[8] Leong PL, Andrews GA, Johnson DE, Dyer KF, Xi S, Mai JC, Robbins PD, Gadiparthi S, Burke NA, Watkins SF, Grandis JR (2003). Targeted inhibition of Stat3 with a decoy oligonucleotide abrogates head and neck cancer cell growth. Proc Natl Acad Sci.

[9] Gao L, Zhang L, Hu J, Li F, Shao Y, Zhao D, Kalvakolanu DV, Kopecko DJ, Zhao X, Xu D-Q (2005). Down-regulation of signal transducer and activator of transcription 3 expression using vector-based small interfering RNAs suppresses growth of human prostate tumor in vivo. Clin Cancer Res.

[10] Zhang X, Zhang J, Wang L, Wei H, Tian Z (2007). Therapeutic effects of STAT3 decoy oligodeoxynucleotide on human lung cancer in xenograft mice. BMC Cancer.

[11] Klosek SK, K-i Nakashiro, Hara S, Goda H, Hamakawa H (2008). Stat3 as a molecular target in RNA interference-based treatment of oral squamous cell carcinoma. Oncol Rep.

[12] Zhao M, Jiang B, Gao FH (2011). Small molecule inhibitors of STAT3 for cancer therapy. Curr Med Chem.

[13] Huang C, Yang G, Jiang T, Cao J, Huang KJ, Qiu ZJ (2011). Down-regulation of STAT3 expression by vector-based small interfering RNA inhibits pancreatic cancer growth. World J Gastroenterol.

[14] Nakanishi C, Toi M (2005). Nuclear factor-[kappa]B inhibitors as sensitizers to anticancer drugs. Nat Rev Cancer.

[15] Welte T, Zhang SS, Wang T, Zhang Z, Hesslein DG, Yin Z, Kano A, Iwamoto Y, Li E, Craft JE, Bothwell AL, Fikrig E, Koni PA, Flavell RA, Fu XY (2003). STAT3 deletion during hematopoiesis causes Crohn’s disease-like pathogenesis and lethality: a critical role of STAT3 in innate immunity. Proc Natl Acad Sci USA.

[16] Lee H, Herrmann A, Deng JH, Kujawski M, Niu G, Li Z, Forman S, Jove R, Pardoll DM, Yu H (2009). Persistently activated Stat3 maintains constitutive NF-kappaB activity in tumors. Cancer Cell.

[17] Han SS, Yun H, Son DJ, Tompkins VS, Peng L, Chung ST, Kim JS, Park ES, Janz S (2010). NF-kappaB/STAT3/PI3K signaling crosstalk in iMyc E mu B lymphoma. Mol Cancer.

[18] Bonner JA, Yang ES, Trummell HQ, Nowsheen S, Willey CD, Raisch KP (2011). Inhibition of STAT-3 results in greater cetuximab sensitivity in head and neck squamous cell carcinoma. Radiother Oncol.

[19] Prêle CM, Keith-Magee AL, Murcha M, Hart PH (2007). Activated signal transducer and activator of transcription-3 (STAT3) is a poor regulator of tumour necrosis factor-alpha production by human monocytes. Clin Exp Immunol.

[20] Grivennikov SI, Karin M (2010). Dangerous liaisons: STAT3 and NF-κB collaboration and crosstalk in cancer. Cytokine Growth Factor Rev.

[21] Quinton LJ, Mizgerd JP (2011). NF-kappaB and STAT3 signaling hubs for lung innate immunity. Cell Tissue Res.

[22] Ellert-Miklaszewska A, Kaminska B, Konarska L (2005). Cannabinoids down-regulate PI3K/Akt and Erk signalling pathways and activate proapoptotic function of Bad protein. Cell Signal.

[23] Li J, Piao YF, Jiang Z, Chen L, Sun HB (2009). Silencing of signal transducer and activator of transcription 3 expression by RNA interference suppresses growth of human hepatocellular carcinoma in tumor-bearing nude mice. World J Gastroenterol.

[24] Chatterjee M, Stühmer T, Herrmann P, Bommert K, Dörken B, Bargou RC (2004). Combined disruption of both the MEK/ERK and the IL-6R/STAT3 pathways is required to induce apoptosis of multiple myeloma cells in the presence of bone marrow stromal cells. Blood.

[25] Chaturvedi MM, Sung B, Yadav VR, Kannappan R, Aggarwal BB (2011). NF-kappaB addiction and its role in cancer: ‘one size does not fit all’. Oncogene.

[26] Qiu S, Adema CM, Lane T (2005). A computational study of off-target effects of RNA interference. Nucleic Acids Res.

[27] Sigoillot FD, King RW (2011). Vigilance and validation: keys to success in RNAi screening. ACS Chem Biol.

[28] Tschuch C, Schulz A, Pscherer A, Werft W, Benner A, Hotz-Wagenblatt A, Barrionuevo LS, Lichter P, Mertens D (2008). Off-target effects of siRNA specific for GFP. BMC Mol Biol.

[29] Burchard J, Jackson AL, Malkov V, Needham RH, Tan Y, Bartz SR, Dai H, Sachs AB, Linsley PS (2009). MicroRNA-like off-target transcript regulation by siRNAs is species specific. RNA.

[30] Jackson AL, Linsley PS (2010). Recognizing and avoiding siRNA off-target effects for target identification and therapeutic application. Nat Rev Drug Discov.

[31] Castanotto D, Rossi JJ (2009). The promises and pitfalls of RNA-interference-based therapeutics. Nature.

[32] Boudreau RL, Monteys AM, Davidson BL (2008). Minimizing variables among hairpin-based RNAi vectors reveals the potency of shRNAs. RNA.

[33] Terasawa K, Shimizu K, Tsujimoto G (2011). Synthetic pre-miRNA-based shRNA as potent RNAi triggers. J Nucleic Acids.

[34] Lee TL, Yeh J, Friedman J, Yan B, Yang X, Yeh NT, Van Waes C, Chen Z (2008). A signal network involving coactivated NF-kappaB and STAT3 and altered p53 modulates BAX/BCL-XL expression and promotes cell survival of head and neck squamous cell carcinomas. Int J Cancer.

[35] Alshamsan A, Hamdy S, Samuel J, El-Kadi AO, Lavasanifar A, Uludağ H (2010). The induction of tumor apoptosis in B16 melanoma following STAT3 siRNA delivery with a lipid-substituted polyethylenimine. Biomaterials.

[36] Alshamsan A, Hamdy S, Haddadi A, Samuel J, El-Kadi AO, Uludağ H, Lavasanifar A (2011). STAT3 knockdown in B16 melanoma by siRNA lipopolyplexes induces bystander immune response in vitro and in vivo. Transl Oncol.

[37] Adach-Kilon A, Swiatek-Machado K, Kaminska B, Dabrowski M (2011). Signal transducer and activator of transcription 1 (Stat1) maintains basal mRNA expression of pro-survival stat3-target genes in glioma C6 cells. J Cell Biochem.

