# Supplementary figures and images for: Off-target effects of plasmid-transcribed shRNAs on NFκB signaling pathway and cell survival of human melanoma cells
Source: Mol Biol Rep. 2013 Oct 30;40(12):6977–86. doi: 10.1007/s11033-013-2817-7 (PMC3835955; doi:10.1007/s11033-013-2817-7)

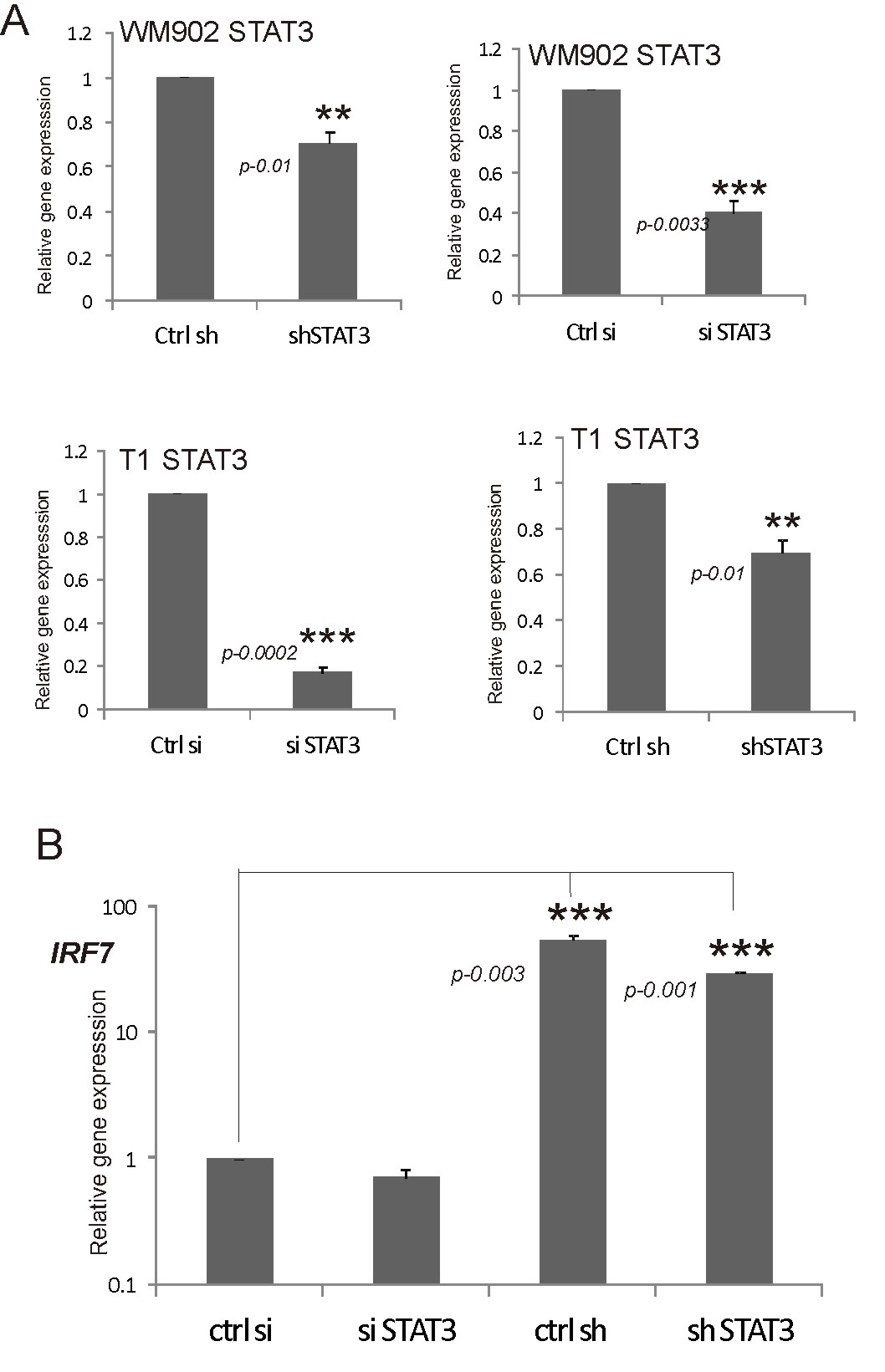

Supplement: Supplementary file 1 — Supplementary Fig. 1. a Level of STAT3 silencing at the level of RNA were determined using qPCR and related to its levels in cells transfected with the control shRNA or siRNA in case of T1 cells. Data are presented as mean ± S.D. from 3 experiments. b Relative expression of IRF7 after transfection of melanoma cells with both siRNA and shRNA against STAT3. Data are presented as mean ± S.D. from 3 experiments. The significant increase of IRF7 expression was observed in cells transfected with shRNAs. (JPEG 147 kb) [file 11033_2013_2817_MOESM1_ESM.jpg]
